# Predictive value of suprasellar extension for intracranial infection after endoscopic transsphenoidal pituitary adenoma resection

**DOI:** 10.1186/s12957-023-03243-y

**Published:** 2023-11-22

**Authors:** Mingjian Lin, Wenbo Wang, Lejian Tang, Yunxiang Zhou, Wencai Li, Jing Xiao, Zhizhu Peng, Xuewei Xia

**Affiliations:** 1 Department of Neurosurgery, GaoZhou People’s Hospital, Gaozhou, 525200 Guangdong China; 2 Department of Neurosurgery, Nanxishan Hospital of Guangxi Zhuang Autonomous Region, Guilin, 541001 Guangxi China; 3 Department of Neurosurgery, Affiliated Hospital of Guilin Medical College, Guilin, 541001 Guangxi China; 4 Department of Neurosurgery, Huizhou Central People’s Hospital, Huizhou, 516000 China

**Keywords:** Pituitary adenoma, Suprasellar extension, Transsphenoidal surgery, Intracranial infection, Predictors

## Abstract

**Objective:**

To investigate the relationship between suprasellar extension (SSE) and intracranial infection after endoscopic endonasal transsphenoidal approach (EETA) for pituitary adenoma resection.

**Methods:**

We retrospectively analyzed 94 patients with suprasellar extended pituitary adenoma admitted to the Department of Neurosurgery of the Affiliated Hospital of Guilin Medical College from January 2018 to December 2021. We measured the preoperative magnetic resonance sagittal SSE and collected clinical data and divided the patients into groups according to the presence of postoperative intracranial infection. The critical value for the SSE was calculated by using a working characteristic curve for the subjects. The risk factors for intracranial infection after EETA resection of pituitary adenomas were analyzed by multivariate regression analysis.

**Results:**

Among the 94 patients, 12 cases (12.8%) were placed in the infection group and 82 cases (87.2%) in the non-infection group. The cut-off value for the SSE in the sagittal position was 15.6 mm, the sensitivity was 75%, the specificity was 87.8%, and the area under the curve (AUC) was 0.801. The coronary cut-off value for the SSE was 15.8 mm, the sensitivity was 66.7%, the specificity was 79.3%, and the AUC was 0.787. The SSE values in the sagittal and coronal positions were correlated with postoperative intracranial infection (*P* < 0.05). After univariate analysis, those with significant differences were included in the multivariate regression analysis. It was concluded that the extension distance of the tumor above the sella in the sagittal position was ≥ 15.6 mm, the tumor texture was hard, and the postoperative cerebrospinal fluid leakage were the independent risk factors for intracranial infection after EETA resection of suprasellar extended pituitary tumors (*P* < 0.05).

**Conclusions:**

The value of SSE on sagittal MRI can predict intracranial infection in patients with suprasellar extended pituitary adenoma after endoscopic endonasal transsphenoidal resection. This finding recommends neurosurgeons pay more attention to the imaging characteristics of pituitary adenomas and select appropriate treatment plans in combination with the intraoperative conditions to reduce the incidence of intracranial infection.

Pituitary adenoma (PA) is a benign tumor occurring in the pituitary fossa, accounting for approximately 15% of intracranial tumors, ranking third in the incidence of intracranial tumors [1]. Among these, suprasellar extension of pituitary adenomas often occurs. In a study, this phenomenon was found in nearly 80% of pituitary adenoma cases [2]. When the pituitary adenoma grows up the sella, it will compress the optic chiasma, resulting in visual impairment and visual field defects, or headaches due to the pressure caused by tumor growth.

Currently, the endoscopic endonasal transsphenoidal approach (EETA) for pituitary adenoma resection is one of the most used surgical methods. Intracranial infection (ICI), however, remains one of the common postoperative complications despite recent advances in endoscopic techniques. ICI will have a serious adverse effect on the prognosis of patients, and may even endanger their lives [3–5]. Previous studies have shown that tumor extension to the sella increases the risk of postoperative pituitary dysfunction and cerebrospinal fluid leakage [6–8], and this leakage is one of the common risk factors of postoperative intracranial infection [9, 10].

There are few studies looking at the factors which affect postoperative intracranial infection from the perspective of imaging when treating suprasellar extension pituitary adenomas, and few studies provide quantitative data on suprasellar extension. In this context, our aim was to study the effect of suprasellar extension distance on postoperative intracranial infection in patients with suprasellar extension pituitary adenomas.

## Data and methods

### Case data

A total of 94 patients with suprasellar extended pituitary adenomas treated at the Affiliated Hospital of Guilin Medical College from January 2018 to December 2021 were selected. All patient clinical data were well recorded (including gender, age, admission symptoms, past medical history, enhanced MRI images before and within 3 days after operation, operation data, and postoperative test results). All patients underwent endoscopic transnasal resection, all performed by the same experienced neurosurgeon.

### Selection and inclusion criteria

**Fig. 1:**
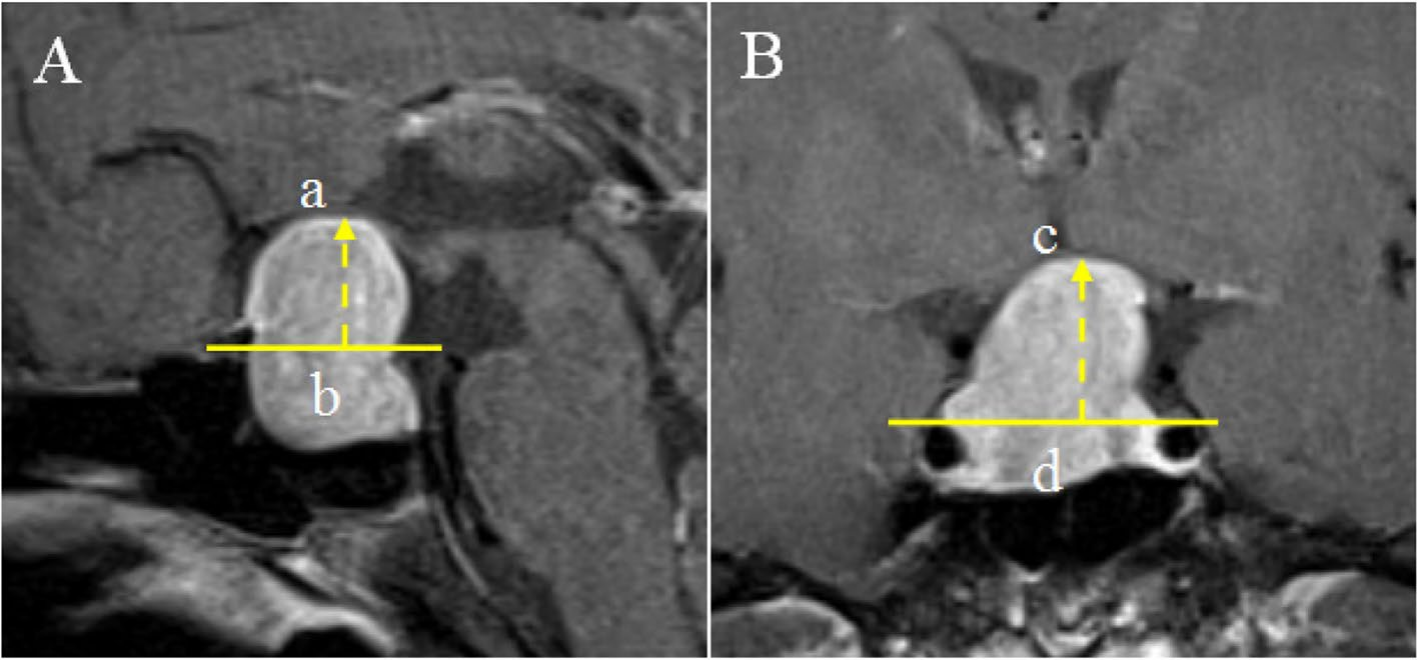
In the sagittal image, draw a reference line between the sellar tubercle and the dorsal sellar, measure the distance from the reference line perpendicular to the highest point of pituitary adenoma, and define the SSE value in the sagittal position (line ab in **A**); In the coronal image, draw a reference line in the horizontal segment of bilateral internal carotid arteries in the cavernous sinus, measure the distance from the reference line perpendicular to the highest point of pituitary adenoma, and define the SSE value in the coronal image (cd line in **B**)

(1) according to findings from imaging examinations, patients with suprasellar extension (Hardy grade ≥ 2) pituitary adenoma were included with reference to Hardy’s pituitary adenoma grading criteria (as shown in Table 1); (2) The SSE values of the pituitary adenomas were measured by sagittal and coronal MRI images. In the sagittal image, a reference line was drawn between the sellar tubercle and the dorsal sellar, and the distance from the reference line perpendicular to the highest point of pituitary adenoma was measured, and the SSE value in the sagittal position was defined (ab line in Fig. 1A); In the coronal image, a reference line in the horizontal segment of bilateral internal carotid arteries in the cavernous sinus was drawn and the distance from the reference line perpendicular to the highest point of pituitary adenoma was measured, and then the SSE value in the coronal image was defined (cd line in Fig. 1B). (3) Diagnostic criteria for postoperative intracranial infection: 1 ~ 4 of the following criteria are clinical diagnosis, as long as the fifth item is met, it can be diagnosed as ICI. If the fifth item is negative, a comprehensive scientific evaluation and diagnosis of the first four items are required. (1) The clinical manifestations include systemic inflammatory reaction, changes in consciousness and mental state, symptoms and signs of increased intracranial pressure, meningeal irritation, and associated symptoms or signs. (2) Blood routine WBC > 10 × 10^9/l, neutrophil ratio > 0.8; (3) Lumbar puncture open pressure > 200 mmH2O, cerebrospinal fluid in the acute stage is turbid, yellow, or purulent; total leukocytes in cerebrospinal fluid > 100 × 10^6/l, neutrophil ratio > 0.7, cerebrospinal fluid glucose content < 2.2 mmol/l, cerebrospinal fluid glucose content/serum glucose content < 0.4; 4) Imaging findings related to intracranial infection. (5) Cerebrospinal fluid specimen smear and culture were positive. (4) Exclusion criteria: (1) patients with intracranial infection and preoperative nasal inflammation caused by meningitis and brain abscess before operation. (2) Patients with other intracranial tumors. (3) There was no cerebrospinal fluid examination record, and the postoperative imaging and/or clinical data were missing.

**Table 1:** Hardy classification criteria for pituitary adenomas

Grade	Description of extension
Grade 1	The tumor diameter is within 10 mm and grows in the saddle
Grade 2	The tumor extends up to 10 mm to the sella and fills the suprasellar cistern
Grade 3	The tumor extends 10 ~ 20 mm to the sella, raising the third ventricle
Grade 4	The tumor extends 20 ~ 30 mm above the sella and compresses the anterior part of the third ventricle
Grade 5	The tumor extended more than 30 mm above the sella and reached the Monro foramen of the lateral ventricle with obstructive hydrocephalus
Grade	Description of invasion
A	Adenoma involving the suprasellar cistern upwards
B	Adenoma extends upwards to the base of the third ventricle, causing it to protrude upwards
C	Adenoma involving the anterior one-third of the third ventricle upwards
D	Asymmetric upward growth, with adenomas protruding into the intracranial cavity and reaching the level of the interventricular foramen
E	Asymmetric lateral growth, invading the cavernous sinus

### Clinical data collection

Detailed information from each patient was recorded, including age, gender, body mass index (BMI), clinical manifestations of admission (vision and visual field change, headache and dizziness, symptoms of abnormal hormone secretion, excessive drinking, polyuria, blepharoptosis, and extraocular muscle paralysis, etc.), history of hypertension and diabetes, history of smoking and alcohol consumption, history of previous craniotomy, hardy classification, operation time (≥ 3 h or < 3 h), the occurrence of cerebrospinal fluid leakage during operation, tumor texture, occurrence of cerebrospinal fluid leakage after the operation (nasal endoscopy after removing nasal packing), presence of intracranial pneumatosis (including pneumatosis in the forehead, operation area, and ventricle) in CT after the operation. In addition, all patients underwent CT or MRI before and after the operation.

### Statistical analyses

SPSS 22.0 statistical software was used for statistical analyses. The measurement data were expressed as mean ± standard deviation (x ± s). If the data conformed to a normal distribution, a *t*-test was used; if not, a rank sum test was used. Chi-square tests were used for counting data. The interobserver reliability was expressed as the intraclass correlation coefficient, also a receiver operating characteristic (ROC) curve was generated to determine the sensitivity and specificity of the sagittal and coronal SSE values in predicting postoperative intracranial infection. When Youden’s index (sensitivity + specificity − 1) reached maximum, a Pearson’s correlation coefficient was used to calculate the correlation between SSE values and postoperative intracranial infection. The statistically significant indices in univariate analysis were included in the multivariate logistic regression model and *P* < 0.05 was statistically significant.

## Results

### Clinical characteristics

A total of 94 patients 44 males (46.8%) and 50 females (53.2%) were included in this study, with an average age of 48 years. Forty-eight (51.1%) patients had a BMI greater than the average of 22.5. Among the 94 patients included in the study, 8 (8.5%) patients had a history of pituitary tumor craniotomy surgery and are now admitted to the hospital for tumor recurrence, 11 (11.7%) patients had a history of hypertension and three (3.2%) had a history of diabetes. As shown in Table 2, The most common admission characteristics were visual impairment and visual field defects (52.1%), followed by headache or dizziness (23.4%), and 10 patients were admitted with abnormal hormone secretion-related symptoms (amenorrhea, lactation, sexual dysfunction, or acromegaly). All patients underwent surgical treatment within 3 days of admission. The total hospitalization time for the infected group was 18.68 ± 5.13 days, while for the non-infected group, it was 9.61 ± 4.56 days. In addition, 32 patients (34.0%) experienced cerebrospinal fluid leakage during the operation, and most patients (63.8%) had soft textures to their tumors. Among the 12 patients with intracranial infection, 7 patients had cerebrospinal fluid leakage and intracranial pneumatosis.

**Table 2:** Main symptoms of hospitalized cases

Main symptoms	Number	Percentage (%)
Vision and visual field changes	49	52.13
Headache and dizziness	22	23.40
Symptoms of abnormal hormone secretion	10	10.64
Other symptoms	13	13.83

### ROC curves in the sagittal and coronal position for predicting postoperative intracranial infection

**Fig. 2:**
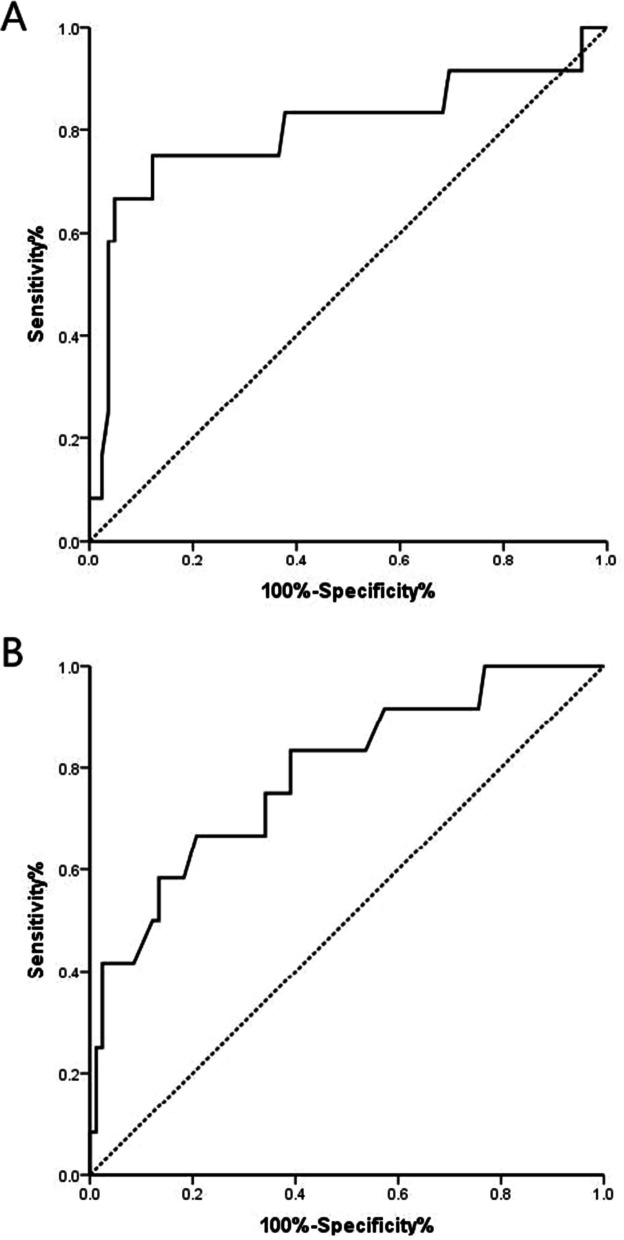
The receiver operating characteristic (ROC) curve was used to evaluate the predictive ability of sagittal (**A**) and coronal (**B**) suprasellar adenoma expansion for postoperative intracranial infection. The areas under the two ROC curves are 0.801 and 0.787 respectively, and the corresponding cut-off values are 15.6 mm and 15.8 mm respectively

According to the working characteristics of the subjects in the ROC curve, the area under the curve (AUC) for sagittal SSE was 0.801 (95% *CI*, 0.629–0.974), and the optimal critical value for the sagittal SSE was 15.6 mm, with a sensitivity of 75% and a specificity of 87.8%; The area under the ROC curve for the coronary SSE value was 0.787 (95%CI, 0.644–0.930). When Youden’s index reached a maximum value, the best cutoff value was 15.8 mm, the sensitivity was 66.7%, and the specificity was 79.3%. See Fig. 2 for details.

### Incidence and treatment of intracranial infection

In this study, 12 patients had intracranial infection after surgery, which made the infection rate 12.8%. For patients with intracranial infection, we monitored the body temperature (BT), white blood cell count (WBC), and C-reactive protein (CRP), and monitored the intracranial infection by lumbar puncture according to the patient’s signs and bloodwork. We used a combination of vancomycin and ceftriaxone for treatment and timely adjusted the antibiotics according to the drug sensitivity results. After the diagnosis of intracranial infection, we immediately performed lumbar cistern drainage to continuously drain cerebrospinal fluid. All patients were cured and discharged without death or permanent defects after treatment.

### Risk factors for intracranial infection

According to univariate analysis (Table 3), the patient gender, operation time, tumor texture, intraoperative cerebrospinal fluid leakage, postoperative cerebrospinal fluid leakage, postoperative intracranial pneumatosis, sagittal, and coronal SSE values all had an impact on the occurrence of postoperative intracranial infection. The results of multivariate logistic regression analysis (Table 4) showed that SSE ≥ 15.6 mm in the sagittal position, hard tumor texture, and cerebrospinal fluid leakage after operation increased the risk of postoperative intracranial infection, which was statistically significant (*P* < 0.05).

**Table 3:** Single-factor analysis of intracranial infection after EETA for suprasellar extension pituitary adenoma surgery

Chateristic		Group		*P* value
		ICI group	NO ICI group	
Sex, *n*(%)				0.036
Male	44 (46.8)	9 (75)	35 (42.7)	
Female	50 (53.2)	3 (25)	47 (57.3)	
Age (mean), *n*(%)				0.751
≥ 48	51 (54.3)	6 (50)	45 (54.9)	
< 48	43 (45.7)	6 (50)	37 (45.1)	
BMI (mean), *n*(%)				0.590
≥ 22.5	48 (51.1)	7 (58.3)	41 (50)	
< 22.5	46 (48.9)	5 (41.7)	41 (50)	
Diabetes mellitus, *n*(%)				1.000
Yes	3 (3.2)	0 (0)	3 (3.7)	
No	91 (96.8)	12 (100)	79 (96.3)	
History of craniotomy, *n*(%)				0.596
Yes	8 (8.5)	2 (16.7)	6 7.3)	
No	86 (91.5)	10 (83.3)	76 (92.7)	
Intraoperative CSF leakage, *n*(%)				0.026
Yes	32 (34.0)	8 (66.7)	24 (29.3)	
No	62 (66.0)	4 (33.3)	58 (70.7)	
Operation time (h), *n*(%)				0.003
≥ 3 h	41 (43.6)	10 (83.3)	31 (37.8)	
< 3 h	53 (56.4)	2 (16.7)	51 (62.2)	
Tumor texture, *n*(%)				0.007
Soft	60 (63.8)	3 (25.0)	57 (69.5)	
Tenacity	34 (36.2)	9 (75.0)	25 (30.5)	
Postoperative CSF leakage, *n*(%)				< 0.001
Yes	12 (12.8)	7 (58.3)	5 (6.1)	
No	82 (87.2)	5 (41.7)	77 (93.9)	
Postoperative intracranial pneumatosis, *n*(%)				0.001
Yes	28 (29.8)	9 (75.0)	19 (23.2)	
No	66 (70.2)	3 (25.0)	63 (76.8)	
Sagittal SSE(mm), *n*(%)				0.004
≥ 15.6 mm	42 (44.7)	10 (83.3)	32 (39.0)	
< 15.6 mm	52 (55.3)	2 (16.7)	50 (61.0)	
Coronal SSE (mm), *n*(%)				0.005
≥ 15.8 mm	43 (46.7)	10 (83.3)	33 (40.2)	
< 15.8 mm	51 (53.3)	2 (16.7)	49 (59.8)

**Table 4:** Multivariate logistic analysis o tension pituitary adenoma surgery

Chateristic	*B*	S.E	Wald	Sig	Exp(*B*)	95% CI
Sex	2.523	1.290	3.822	0.051	12.464	0.994 ~ 156.324
Operation time	1.649	1.204	1.875	0.171	5.201	0.491 ~ 55.104
Tumor texture	− 2.477	1.222	4.108	0.043	0.084	0.008 ~ 0.922
Intraoperative CSF leakage	0.114	1.072	0.011	0.915	1.121	0.137 ~ 9.169
Postoperative CSF leakage	2.814	1.325	4.509	0.034	16.669	1.242 ~ 223.737
Postoperative intracranial pneumatosis	1.617	1.077	2.509	0.133	5.038	0.610 ~ 41.609
Sagittal SSE ≥ 15.6 mm	4.133	2.087	3.921	0.048	62.342	1.043 ~ 3726.914
Coronal SSE ≥ 15.8 mm	− 1.248	1.551	0.647	0.421	0.287	0.014 ~ 5.998

## Discussion

The sellar septum is an extension of the dura mater and a barrier between the contents of the sella and the suprasellar region [11]. The suprasellar extension will mechanically destroy the structure of the sellar septum. Once the suprasellar extension of pituitary adenomas is located in the dura mater and connected with the skull cavity, it will increase the incidence of surgical complications [12].

Intracranial infection is a significant postoperative complication following the endoscopic transsphenoidal resection of pituitary adenomas. Prior investigations have consistently indicated that cerebrospinal fluid leakage represents one of the independent risk factors for this complication, a finding congruent with the outcomes of the present study [13–15]. The incidence of cerebrospinal fluid leakage subsequent to endoscopic management of pituitary adenomas typically falls within a range of 0.5 to 14% [16]. In our cohort, the observed leakage rate was notably higher at 12.8%, placing it at the upper end of this spectrum. This escalated risk can be attributed to the suprasellar extension of the pituitary adenomas, which amplifies the likelihood of cerebrospinal fluid leakage. Huang X et al. [17] have elucidated that postoperative cerebrospinal fluid leakage might serve as a conduit for bacteria to access the brain tissue, thereby instigating intracranial infection. This breach in the brain's protective barriers establishes a potential pathway for external pathogens to precipitate intracranial infections. Moreover, our findings are corroborated by studies that have established intracranial pneumatosis (defined as a maximum pneumatosis cavity diameter of ≥ 1 cm) as an additional independent risk factor for intracranial infection subsequent to endoscopic transsphenoidal pituitary adenoma resection [18].

However, in the multifactorial model from this study, intracranial pneumatosis did not reach statistical significance. Among the 12 patients with intracranial infection after the operation, seven patients had cerebrospinal fluid leakage and intracranial pneumatosis at the same time after the operation, two patients had no cerebrospinal fluid leakage but had intracranial pneumatosis after CT reexamination and both of the two patients were found to have cerebrospinal fluid leakage during operation. The remaining three patients did not have cerebrospinal fluid leakage and intracranial pneumatosis after operation, This indicates that postoperative intracranial pneumatosis is not equal to the presence of cerebrospinal fluid leakage at the same time. We believe that the formation of postoperative intracranial gas accumulation may be due to the channel presence between the brain and the outside world caused by cerebrospinal fluid leakage during the operation, causing gas to enter the brain. However, this channel is transient and after the operator reconstructs and repairs the skull base, the brain can become a closed space again, thus reducing the incidence of postoperative cerebrospinal fluid leakage. In addition, studies have pointed out that when the tilt angle of head elevation is greater than 30°, it can lead to intracranial negative pressure [19]. Among them, sitting/standing posture is more likely to lead to a decrease in intracranial pressure and even negative ICP values, thereby increasing the risk of pneumocranial and cerebrospinal fluid leakage [20–22]. Therefore, in our study, all patients were placed in a half-sitting position after surgery, while avoiding sudden changes in body position. After conservative treatment, 9 patients with postoperative cerebrospinal fluid leakage experienced no symptoms of nasal leakage within one week after surgery; 2 patients require lumbar cistern drainage to release cerebrospinal fluid and accelerate the healing of the leak; Another patient needs to undergo another transnasal cerebrospinal fluid leak repair surgery.

In addition, pituitary adenomas can invade the suprasellar region through the sellar septal foramen during the growth process [11]. For pituitary adenomas with Hardy grade ≥ 2, studies have reported that patients with adenomas extending to the suprasellar region are more likely to have complications such as visual field defect, pituitary dysfunction, cerebrospinal fluid leakage when compared to patients with adenomas extending in other directions [17, 23]. However, there are relatively few studies investigating complications of postoperative intracranial infection. In our study, tumor features were described quantitatively, and pituitary adenomas were measured quantitatively from sagittal and coronal MRI images. Of course, the tumor can also extend to the parasellar and infrasellar regions, but we believe that the extension distance above the sellar region is more important. However, the extension of the tumor to the infrasellar region will reduce the pressure in the sellar region to a certain extent. If the tumor invades the parasellar cavernous sinus, the internal carotid arteries on both sides will be pushed and displaced, and the positions measured on the coronal images will change accordingly. The suprasellar distance is measured based on bone structure in the sagittal position. Therefore, SSE in the sagittal position is relatively constant and more reliable [24].

In our study, preoperative sagittal SSE ≥ 15.6 mm and tumor toughness were independent risk factors for postoperative intracranial infection in patients with suprasellar extended pituitary adenomas. The sellar septum is the dura extension of the skull base, covering the pituitary fossa. The extension and invasion of pituitary adenomas to the sellar septum will break through the sellar septal foramen and destroy the original structure of the sellar septum. For pituitary adenomas with suprasellar extension, the operator needs to remove the tumor to the suprasellar region, which may damage the sellar septum structure again, causing intracranial communication with the outside world, leading to the occurrence of postoperative intracranial infection. The tumor texture will affect the incidence of postoperative foramen leakage [16], further increasing the incidence of postoperative intracranial infection.

During surgery, soft tumors can be easily removed by suction, while tough tumors require difficult surgical procedures, which increases damage to normal tissue. In this study, 10 (83.3%) patients with intracranial infection had tumors with a suprasellar extension distance greater than 15.8 mm. Higher tumor location especially invasion to the suprasellar cistern contributes to a higher risk of intraoperative cerebrospinal fluid leak. Cerebrospinal fluid leakage during operation was also included, but there was no correlation between this factor and postoperative intracranial infection. We believe that in some cases of postoperative cerebrospinal fluid leakage without intraoperative cerebrospinal fluid leakage, the operator needs to evaluate whether there is clearly visible leakage during the operation, which may cause some low-flow cerebrospinal fluid leakage to be ignored, or the sellar septum to collapse due to sneezing after the operation, resulting in leakage. Therefore, some studies suggest that even if cerebrospinal fluid leakage is not found, if there is an obvious subarachnoid hernia, sellar floor reconstruction can be performed [25]. In our research, Low to medium-flow cerebrospinal fluid leakage is covered with artificial dura mater to cover the defect dura mater, accompanied by the application of a fibrin glue to seal the periphery. A thin layer of gelatin sponge was then placed. For high-flow cerebrospinal fluid leaks, artificial dura was introduced, and autologous fat was positioned underneath the dura mater. Defects in the dura mater were reinforced with a fascia lata, free nasal mucosal flaps, or artificial dura. A thin layer of fibrin glue is then sprayed over to ensure these tissues adhere to the skull base, and the nasal cavity is finally filled with an expandable sponge.

## Study limitations

This study has some limitations. First, this was a single-center study with a small sample size (*n* = 94), which may have resulted in bias and inaccuracy in selection. When measuring tumor data, some suprasellar pituitary adenomas are large, leading to the displacement or change of the original structure around the tumor and therefore, the data measured and collected may have errors. Second, the manuscript was designed to clarify the predictive value of the expansion distance of suprasellar tumors for intracranial infections, and there was no control group to indicate that suprasellar expansion increases the risk of intracranial infections.

## Conclusions

In our study, we found that the incidence of intracranial infection after endoscopic transsphenoidal resection of suprasellar extended pituitary adenomas can be predicted by SSE measured by MRI at the sagittal position. In addition, independent risk factors include cerebrospinal fluid leakage and tough tumor texture. Thus, our results remind surgeons to pay more attention to the structural characteristics of pituitary adenomas during the perioperative period, which may be helpful for clinical treatment and surgical decision-making.

## Data Availability

The datasets used and/or analyzed during the current study are available from the corresponding author on reasonable request.

## Abbreviations

EETA: Endoscopic endonasal transsphenoidal approach
SSE: Suprasellar extension
AUC: Area under the curve
PA: Pituitary adenoma
ICI: Intracranial infection
MRI: Nuclear magnetic resonance imaging
BMI: Body mass index
BT: Body temperature
WBC: White blood cell count
CRP: C-reactive protein
